# Molecular crosstalk between apoptosis and autophagy induced by a novel 2-methoxyestradiol analogue in cervical adenocarcinoma cells

**DOI:** 10.1186/1475-2867-13-87

**Published:** 2013-08-27

**Authors:** Anne E Theron, Elsie M Nolte, Laurence Lafanechère, Annie M Joubert

**Affiliations:** 1 Department of Physiology, Faculty of Health Sciences, University of Pretoria, Private Bag X323, Arcadia, 0007 Gauteng, Pretoria, South Africa; 2 Department of Cellular Differentiation and Transformation, Team# 03: Polarity, Development and Cancer, Université Joseph Fourier, Albert Bonniot Institute, CRI INSERM/UJF U823, Grenoble, France

**Keywords:** 2-methoxyestradiol, ESE-16, Analogue, Apoptosis, Autophagy, Caspase, Crosstalk, Mitotic block, Microtubules, 2-ethyl-3-*O*-sulphamoyl-estra-1,3,5(10)16-tetraene

## Abstract

**Background:**

2-Methoxyestradiol has been shown to induce both autophagy and apoptosis in various carcinogenic cell lines. Although a promising anti-cancer agent, it has poor bioavailability and rapid *in vivo* metabolism which decreases its efficiency. In order to improve 2-methoxyestradiol’s anti-proliferative properties, a novel 2-methoxyestradiol analogue, 2-ethyl-3-*O*-sulphamoyl-estra-1,3,5 (10)16-tetraene (ESE-16), was previously *in silico*-designed in our laboratory. This study investigated ESE-16 for its anti-proliferative potential on a cervical adenocarcinoma cell (HeLa) cell line. Additionally, the possible intracellular crosstalk mechanisms between the two types of cell death were investigated.

**Methods and results:**

HeLa cells exposed to 0.5 μM ESE-16 for 24 hours showed morphological evidence of both apoptotic and autophagic death pathways as assessed by polarization-optical transmitted light differential interference contrast microscopy, fluorescent microscopy and transmission electron microscopy. Flow cytometric cyclin B1 quantification revealed induction of programmed cell death after halting cell cycle progression in metaphase. Confocal microscopy demonstrated that ESE-16 caused microtubule fragmentation. Flow cytometric analysis of cell cycle progression and phosphatidylserine flip determination confirmed induction of apoptosis. Moreover, an increase in aggresome formation and microtubule-associated protein light chain, LC3, was demonstrated indicative of autophagy. Both caspase 8 and 3 were upregulated in a spectrophotometric analysis, indicating the involvement of the extrinsic pathway of apoptotic induction.

**Conclusions:**

We conclude that the novel *in silico*-designed compound, ESE-16, exerts its anti-proliferative effect on the tumorigenic human epithelial cervical (HeLa) cells by sequentially targeting microtubule integrity, resulting in a metaphase block, causing induction of both autophagic and apoptotic cell death via a crosstalk mechanism that involves the extrinsic pathway. Future investigations will expand on signal transduction pathways involved in both apoptosis and autophagy for assessment of ESE-16 effects on microtubule dynamic instability parameters.

## Background

**Figure 1:**
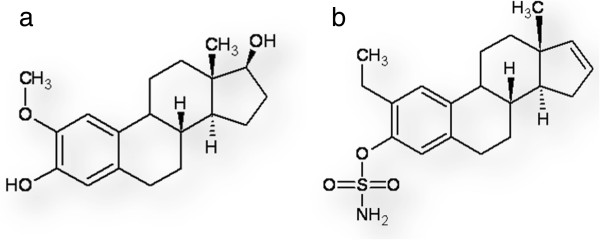
**Structure of 2-ME and ESE-16. (a)** shows the parent compound, (17 beta)-2-methoxyestra-1,3,5(10)-triene-3,17-diol (2-methoxyestradiol). **(b)** indicates the substitutions at position 2, 3 and 17 of 2-ethyl-3-*O*-sulphamoyl-estra-1,3,5(10)16-tetraene (ESE-16), a sulphamoylated 2-ME analogue [33].

Registered as Panzem® by Entremed, Inc (Rockville, MD), 2-methoxyestradiol (2-ME) (Figure 1a), an endogenous metabolite of 17β-estradiol resulting from sequential hepatic hydroxylation and methylation by cytochrome P450 enzymes and catechol-*O*-methyltransferase respectively, has undergone phase II clinical trials [1-3]. 2-ME induces apoptosis in endothelial cells, as well as cancer cells by the intrinsic and extrinsic pathways by both caspase dependent and -independent pathways [2,4-6]. Not only has 2-ME proven effective in primary treatment settings, but it has also demonstrated the capability to sensitize therapy-resistant neoplastic cell types to conventional anti-cancer treatments including radiation therapy [7,8]. For this reason 2-ME seemed to be a promising anti-cancer agent, especially in the light of findings that non-neoplastic cell lines were less affected than cancer cell lines by exposure to the compound [9].

Pharmacodynamics of 2-ME appeared to be desirable in the treatment of neoplasms, but the pharmacokinetic picture proved problematic. 2-ME has a short half-life and a poor bioavailability. Being a target for 17β-hydroxysteroid dehydrogenase-mediated metabolism [10-13], 2-ME is metabolized with conjunction at the 3 and 17 positions, together with oxidation at the 17 position [10]. Strategies have been considered to overcome this rapid biodegradation and to improve its bioavailability. These strategies include the design of nanocrystal dispersion techniques (such as Panzem NCD^TM^) [3], finding new methods of delivery such as encapsulation [14] and the design of 2-ME analogues [15-20].

The design of 2-ME analogues aimed to enhance the already present anti-mitotic and anti-angiogenic properties of 2-ME itself [20,21]. Dehydration at the metabolically active 17 position allowed retention of cytotoxic and anti-tubulin characteristics together with a decreased metabolic breakdown [22,23]. When combined with a substitution at position 3 to increase potency, 3-carboxyamide-2-methoxyestra-1,3,5 (10)16-tetraene demonstrated suitable anti-mitotic and anti-microtubule properties with an improved pharmacokinetic profile *in vitro* and *in vivo*[22].

Sulphamate substitutions on estrogen molecule analogues increase the estrogenic bioavailability due to avoidance of the first hepatic bypass metabolism [24]. This is attributed to the sulpha moieties’ ability to reversibly bind to carbonic anhydrase II (CAII) in erythrocytes, followed by a slow release into the plasma [24,25]. CAIX is over-expressed in the immediate tumor environment giving neoplastic cells a growth advantage in their acidic and hypoxic extracellular milieu [26,27]. CAs also promote extracellular acidic activation of the metalloproteinases thereby enhancing the neoplastic cell invasiveness [28]. Therefore selective inhibition of CAIX provides a promising mechanism to manipulate the extracellular tumor milieu and to curtail metastatic tendencies [27].

2-Methoxyestradiol-3,17-O,O-bis-sulfamate (2-MEbisMATE) is a 2-ME analogue, which, by adding a sulpha moiety at position 3, fulfills two objectives: increasing the anti-mitotic and spindle disruption capacities (position 3 modification) and increasing the bioavailability with the sulpha group [11,29-32]. Literature reported a more potent anti-mitotic (10-fold), anti-spindle and anti-angiogenic (60-fold) effect of 2-MEbisMATE when compared to 2-ME *in vitro* and *in vivo*[11,29,32].

Although 2-MEbisMATE has a positive profile of anti-cancer and anti-angiogenic activities, potency and specificity in CAIX binding needed to be improved. Stander *et al.* designed a range of 3 sulphamoylated 2-ME analogues *in silico* by modifying the 2- and 17 positions with moieties known to improve the anti-mitotic activity and to increase the compounds’ half-life [33]. The designed compounds were analyzed using a molecular modeling simulation and docking software, namely AutoDockTools4, to determine the best binders to the tubulin colchicine binding site and to CAIX [33].

2-Ethyl-3-*O*-sulphamoyl-estra-1,3,5(10)16-tetraene (ESE-16) (Figure 1b) is one of the novel *in silico-*designed sulphamoylated 2-ME analogues that needs still to be fully investigated for its potential anti-proliferative characteristics. ESE-16 is not currently commercially available. *In silico* modeling analysis indicated a significant preference of CAIX over CAII binding, presenting a method to deliver the drug more specifically to acidic tumor environments. Our laboratory has established the anti-proliferative action of ESE-16 at nanomolar concentrations in cell lines including MCF-7 breast cancer cells (estrogen-receptor positive), metastatic MDA-MB-231 breast cancer cells, non-tumorigenic MCF-12A breast cells and SNO non-keratinizing squamous epithelium cancer [33].

This study aimed to determine whether the novel *in silico*-designed 2ME analogue, ESE-16, retained the previously described anti-proliferative properties of the parent compound *in vitro*. Since the compound was designed to bind to the colchicine site of the microtubules, the effect of ESE-16 on the spindle integrity was determined. Effect of this spindle disruption on cell cycle progression and resultant induction of programmed cell death pathways were investigated.

## Results

### Cell cycle

**Figure 2:**
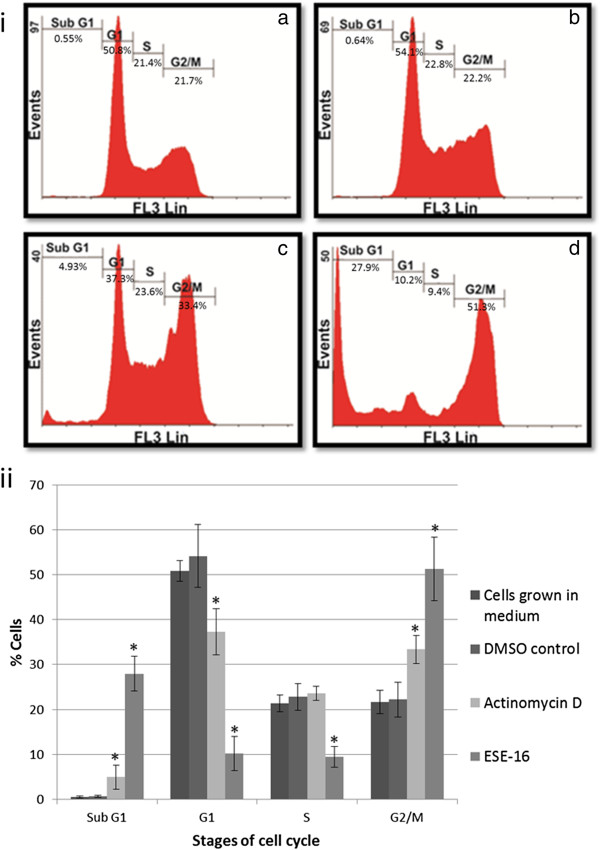
**Cell cycle analysis after 24 hour exposure to ESE-16. (2i)** Histograms derived from flow cytometry comparing cell distribution within the cell cycle between control samples and ESE-16 treated cells. **(2ia)** Cell cycle distribution of cells grown in medium only. **(2ib)** Cells exposed to DMSO as a vehicle control. **(2ic)** Cells exposed to actinomycin D as a positive apoptosis control. **(2id)** Cells exposed to ESE-16 demonstrating an increase in G_2_/M and sub-G_1_ populations with a concurrent decrease in G_1_- and S phase. **(2ii)** Graphical representation of the histogram results. (* indicates a statistically significant difference when compared to DMSO vehicle control samples with a *P* value of < 0.05). Standard deviation is indicated by T-bars.

Flow cytometry was used to determine the effect of ESE-16 on the progression of exposed cells through the cell cycle, thus indicating an influence on cell viability. Results were calculated as percentage cells in each stage of the cell cycle. Cell cycle distribution of the cells propagated in growth medium (negative control) revealed an average of 50.82 ± 2.36% in the G_1_ phase, 21.41 ± 1.85% in the S phase and 21.73 ± 2.61% in the G_2_/M phase (Figure 2ia). Only 0.55 ± 0.19% of the cells were present in sub-G_1_ population. There was no statistically significant difference between cells propagated in growth medium (negative control) and the DMSO vehicle control sample, both representing a cell population in logarithmic growth (Figure 2ib). Cell cycle distribution changed significantly with actinomycin D- and ESE-16 exposure. A statistically significant increase of cells in sub-G_1_ was demonstrated in the actinomycin D sample (4.93 ± 2.7%, *P* = 0.04) (Figure 2ic) and the ESE-16-treated cells (27.93 ± 3.86%, *P* = 0.0002) (Figure 2id). G_1_ populations were significantly decreased, to 37.29 ± 5.08% (*P* = 0.028) and 10.16 ± 3.79% (*P* = 0.0007) of the cell population in actinomycin D- and ESE-16-treated cells respectively. ESE-16 showed a decrease in viable cells (G_1_- and S phases) and a distinct increase of cells in G_2_/M phase (51.28 ± 7.09%, *P* = 0.0034). Figure 2ii summarizes the flow cytometric data of the cell cycle comparing the distribution of cells within the cell cycle in the various samples.

### Apoptosis studies

**Figure 3:**
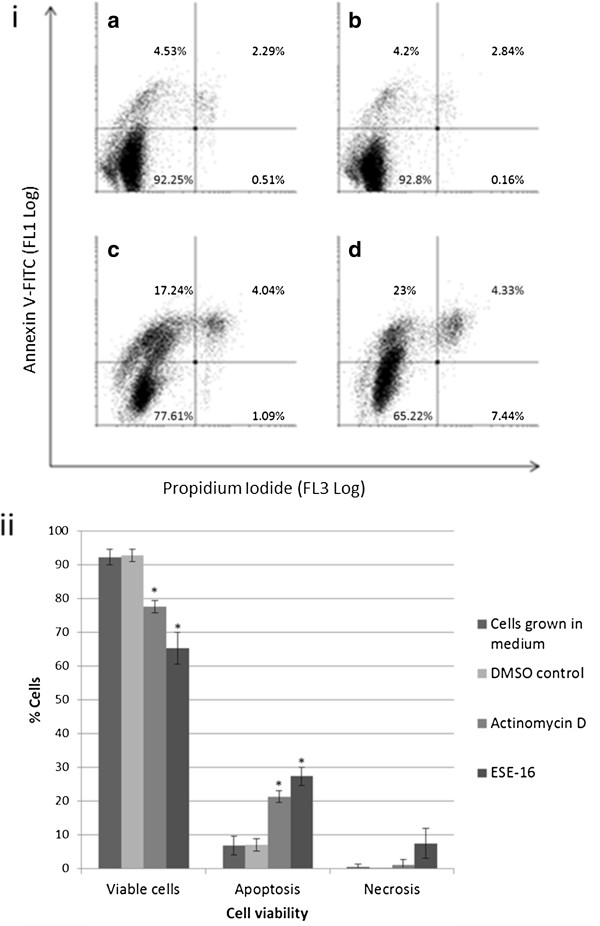
**Analysis of HeLa cell viability after 24 hour ESE-16 exposure.** Apoptosis was determined by flow cytometric quantification of phosphatidylserine-flip. **(3i)** Dots plots of HeLa cells after 24 hour exposure to growth medium **(3ia)**, DMSO vehicle control **(3ib)**, actinomycin D positive apoptosis control **(3ic)** and ESE-15 **(3id)**. A statistically significant decrease of viable cells was seen in actinomycin D- and ESE-16-exposed cells with a concurrent increase of apoptotic cells. There were no statistically significant differences observed between cells propagated in growth medium only and DMSO vehicle controls in any of the cell cycle categories. No significant difference was calculated in the amount of necrotic cells in all samples. **(3ii)** Graphical representation of the dot plot data (* *P* < 0.05, standard deviation represented by T-bars).

In order to detect and to discriminate between the induction of apoptosis, necrosis and viable cells, flow cytometric analysis using labeled annexin antibodies for the detection of phosphatidylserine flip in ESE-16-exposed cell membranes was done. Analysis of the data generated from dot plots (Figure 3i) via Cyflogic version 1.2.1 software revealed that in a healthy cell population (DMSO as a vehicle control), 92.8 ± 1.84% of the cells were viable, with 7.02 ± 1.83% undergoing apoptosis and an insignificant 0.16 ± 0.02% in necrosis. There were satistically insignificant differences between cells propagated in growth medium and DSMO vehicle controls. Actinomycin D induced apoptosis (21.28 ± 1.76%, *P* = 0.0006), with a significant decrease of viable cells (77.68 ± 1.89%, *P* = 0.0005). A decrease in cell viability was demonstrated in the ESE-16-exposed cells (64.22 ± 4.67%, *P* = 0.0006), with a corresponding rise in apoptotic cell numbers (27.32 ± 2.67%, *P* = 0.0004). There was no significant difference in the proportion of necrotic cells between the samples (*P* = 0.08). Results are summarized graphically in Figure 3ii.

### Cyclin B1 detection

**Figure 4:**
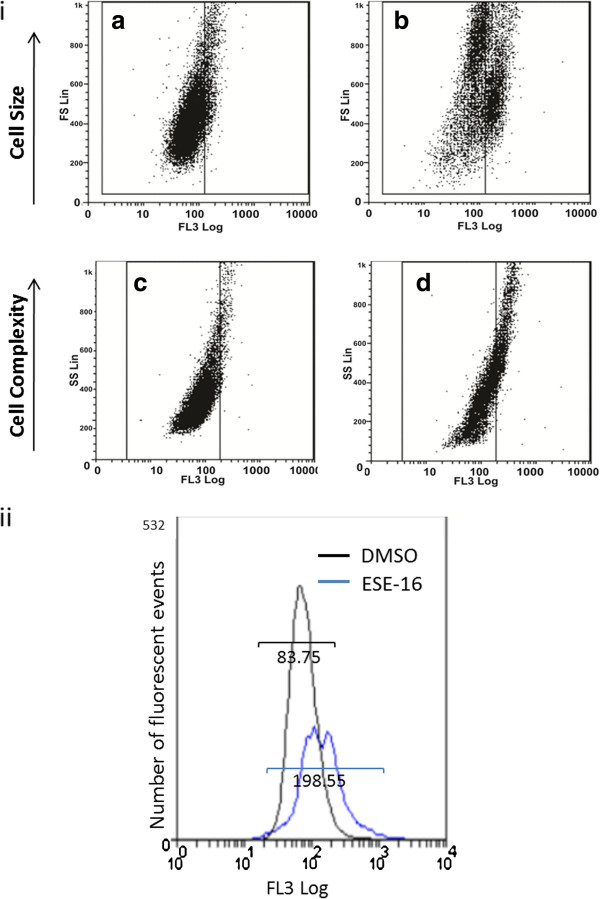
**Flow cytometric evaluation of cyclin B1 up-regulation in HeLa cells. (4i)** Dot plots of ESE-16 exposed cells **(b and d)** were compared to the DMSO vehicle control **(a and c)**. FL3 log fluoresce intensity was plotted against cell size determined by forward scatter **(a and b)** and cell complexity as established with side scatter **(c and d)**. An increase in expression of cyclin B1 in the ESE-16-treated cells was observed in the smaller **(b)** and more complex **(d)** cells. **(4ii)** Intensity histogram of cyclin B1 expression within the DMSO vehicle control (black) and ESE-16-exposed cells (blue) demonstrating an increase in intensity and a right shift in the drug-exposed cells.

A cyclin B1-phycoerythrin conjugated antibody (Milli-Mark™ Anti-Cyclin B1-PE, clone GNS3 (8A5D12)) was used to quantify cyclin B1 protein up-regulation in ESE-16-treated HeLa cells by employing flow cytometric analyses. This was conducted to determine the compound’s ability to induce a metaphase block. Figure 4i displays representative scatter plots, with the fluorescent intensity of the PE-conjugated cyclin B1 antibody plotted against forward scatter as a determinant of cells size (Figure 4ia and ib) and side scatter indicative of cell complexity (Figure 4ic and id). Scatter plots displayed a higher fluorescent signal in more complex and smaller sized cells which are representative of cells in metaphase [34,35]. Figure 4ii shows the intensity histogram, demonstrating an increased intensity and a right shift of the ESE-16-exposed cells. A 2.47 ± 0.49-fold increase of cyclin B1 expression (*P* < 0.05) in the ESE-16-exposed cells was revealed when compared to the DMSO vehicle control.

### Aggresome detection: Autophagy

**Figure 5:**
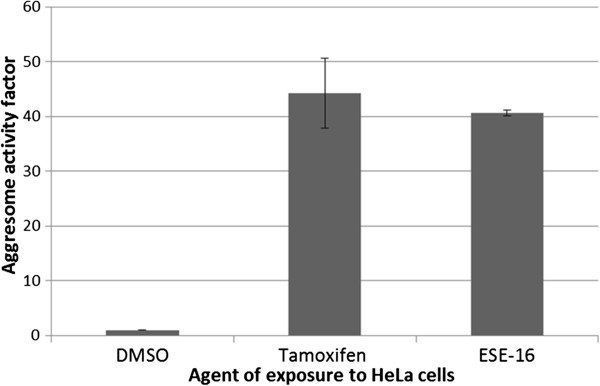
**Aggresome activity factor in ESE-16-exposed HeLa cells.** Cells treated with tamoxifen and ESE-16 was compared to the negative DMSO vehicle control. Standard deviations are annotated with T-bars. An AAF > 25 represents a significant increase in intracellular aggresome formation, strongly indicative of autophagy (standard deviation represented by T-bars).

In order to evaluate the induction of autophagy in ESE-16-treated cells, flow cytometric quantification of aggresome formation was conducted. Using the formula provide in the manufacturer’s protocol, the aggresome activity factor (AAF) was calculated. According to the manafacture’s protocol, an AAF of more than 25 is regarded as a statistically significant increase in aggresome formation. There was no statistically significant difference between cells propagated in growth medium and DMSO vehicle control samples. Figure 5 is a graphic representation of the AAF of tamoxifen- and ESE-16-treated cells, calculated at 44.3 ± 6.36 and 40.7 ± 0.53 repectively. Data indicate that ESE-16 induces aggressome formation, indicative of autophagy.

### Autophagy-related protein LC3 B determination

**Figure 6:**
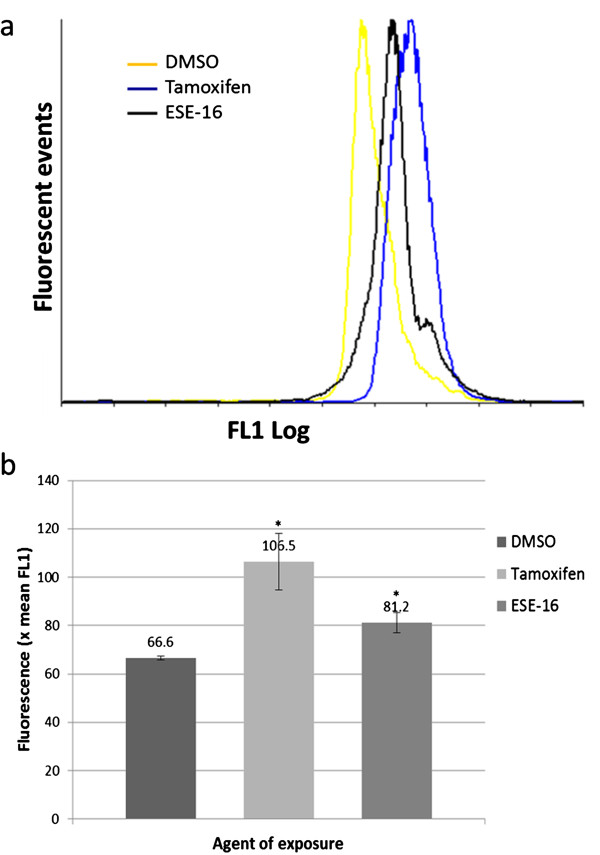
**Autophagy-related protein LC3 B determination in ESE-16-exposed HeLa cells. (a)** Overlay histogram of LC3 B expression in HeLa cells exposed to the DMSO vehicle control (yellow), tamoxifen (blue) and ESE-16 (black), demonstrating a right shift in the treated samples. **(b)** Graphic representation of the increase in LC3 B fluorescence between the DMSO vehicle control and ESE-16-exposed HeLa cells (* *P* < 0.05, standard deviation represented by T-bars).

Flow cytometric analysis of autophagy-related protein LC3 B expression was done to quantify the formation of autosomal vacuoles in ESE-16-eposed HeLa cells. The MAP1LC3 B rabbit antibody (Biosensis, Thebarton, Australia) was utilized and the FL1 fluorescence of at least 10 000 cells were analyzed per repeat. There was a statistically significant increase of LC3 B within the tamoxifen (x mean FL1 = 106.5 ± 11.6; *P* = 0.041) and ESE-16-exposed cells (x mean FL1 = 81.2 ± 4.08; *P* = 0.040) when compared to the DMSO vehicle control (x mean FL1 = 66.6 ± 0.82) (Figure 6).

### Signal transduction: quantification of caspases 8 and 3

**Figure 7:**
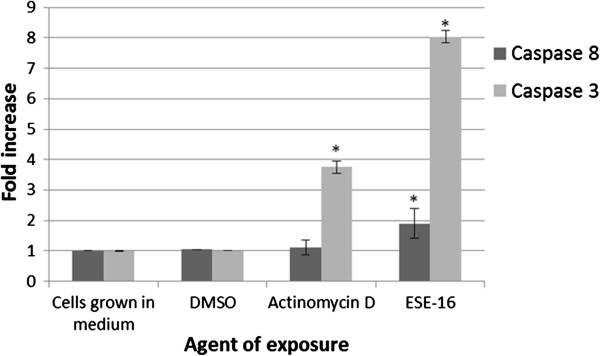
**Fold increase in caspase 8 and 3 activity in ESE-16-treated cells.** Compound treated cells were compared to cells propagated in growth medium (MO), the DMSO vehicle and actinomycin D controls (* *P* < 0.05, standard deviation represented by T-bars).

The FLICE/Caspase 8 colorimetric kit was used to determine caspase 8 activation in cell lysates of the ESE-16 exposed HeLa cells and of the relevant controls. There was no statistically significant difference noted between readings of cells propagated in growth medium only, DMSO vehicle and actinomycin D controls. A 1.9 ± 0.4-fold increase in caspase 8 induction in the ESE-16-exposed HeLa cells was observed when compared to the baseline (*P-*value 0.04) (Figure 7). Caspase 3 activity in cell lysates was measured using the BioVision Caspase-3/CPP32 Colorimetric Assay Kit. Cells propagated in growth medium and DMSO vehicle controls showed no statistical difference between these results. A statistically significant up-regulation of caspase 3 activity in the actinomycin D positive control (3.75 ± 0.21-fold increase, *P* = 0.011) was noted and a pronounced increase in the ESE-16-treated cells (8.03 ± 0.21-fold increase, *P* = 0.008) (Figure 7).

### Polarization-optical transmitted light differential interference contrast microscopy (PlasDIC)

**Figure 8:**
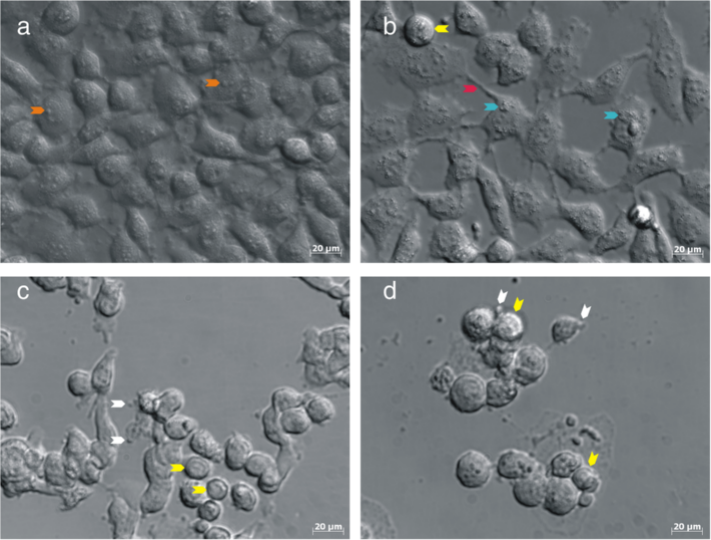
**PlasDIC micrographs of HeLa cells after a 24 hour exposure. (a)** DMSO (vehicle control) exposure demonstrated confluent growth of HeLa cells. Cells were mostly in interphase. **(b)** Tamoxifen exposure (positive autophagy control) demonstrated a reduction in cell density and an increase in vacuolar structures and cell protrusions (cell distress). **(c)** Actinomycin D exposure (positive apoptosis control) resulted in a reduction in cell density, apoptotic bodies, shrunken cells, ghost cells and cellular debris. **(d)** HeLa cells exposed to 0.5 μM ESE-16, demonstrating the hallmarks of metaphase block and apoptosis. (Arrow colour key: orange = nucleoli; blue = vacuolar structures; yellow = rounded cells in metaphase; red = cell protrusion; white = apoptotic bodies).

To substantiate flow cytometric data and to evaluate ESE-16’s anti-proliferative effects, PlasDIC microscopy was used. Cells exposed to DMSO as a vehicle control (v/v%) (Figure 8a) continued to grow in a logarithmic manner resulting in confluent cells in various stages of mitosis (mostly interphase). Cells treated with tamoxifen revealed increased presence of vacuolar structures (most likely to be autophagosomes) and cells in distress (cell protrusions) (Figure 8b). Cells exposed to actinomycin D revealed apoptotic bodies, multiple shrunken cells, ghost cells and scattered cell debris (Figure 8c). Shrunken and round cells in metaphase block, the formation of apoptotic bodies, as well as evidence of cell debris were visible in the ESE-16-treated HeLa cells (Figure 8d). All treated samples displayed a decrease in cell density.

### Fluorescent microscopy

**Figure 9:**
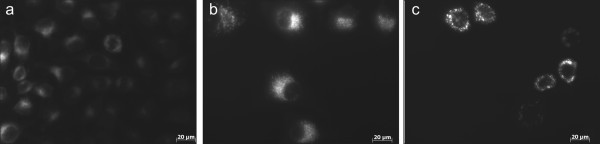
**MDC fluorescence microscopy of ESE-16-exposed HeLa cells. (a)** DMSO (vehicle control) demonstrated dividing cells with satisfactory confluency and non-specific MDC staining. **(b)** Tamoxifen-exposed cells demonstrating MDC-stained vacuoles. **(c)** ESE-16-exposed cells demonstrated a compromised cell density and the formation of MDC-stained vacuoles.

In order to substantiate autophagy induction, a fluorescent microscopic technique using monodansylcadaverine (MDC) was employed as described by Laane *et al.*[36]. Figure 9a is a representative image of HeLa cells exposed to DMSO as a vehicle control which displayed confluent growth and non-specific MDC staining. Cells exposed to tamoxifen as the positive autophagy control demonstrated the formation of MDC-positive vacuoles (Figure 9b), as did the ESE-16-exposed cells (Figure 9c). A UV filter with excitation 380 nm and emission 420 nm was used for MDC staining detection.

### Transmission electron microscopy

**Figure 10:**
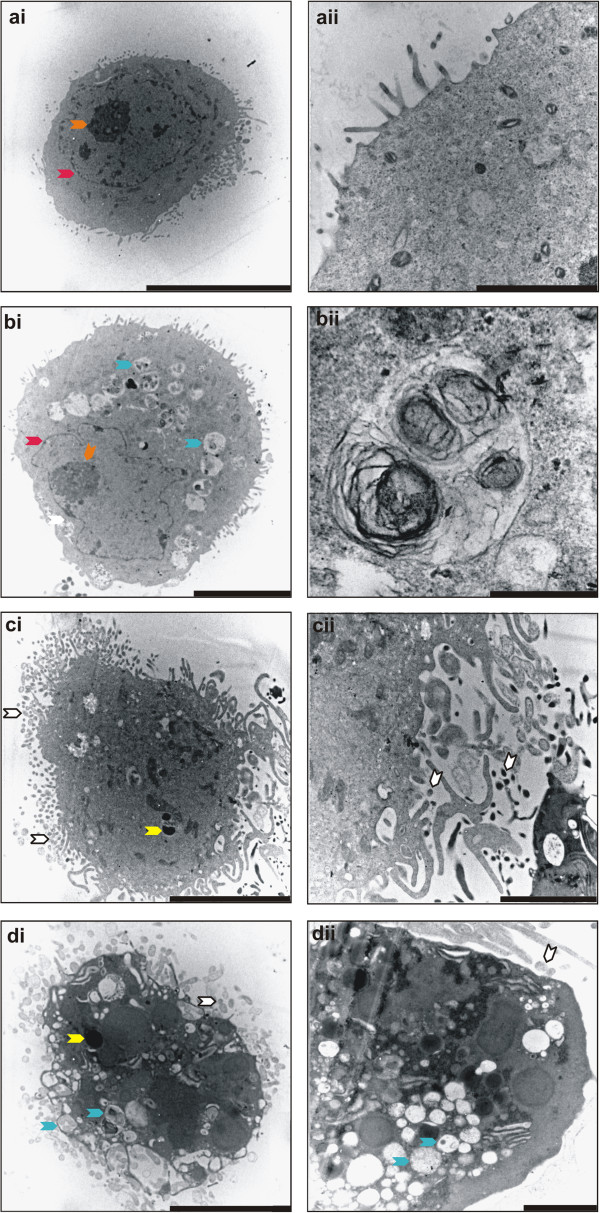
**Transmission electron microscopy of exposed HeLa cells. (ai)** HeLa cell exposed to the DMSO vehicle control which demonstrated a smooth well-defined cell membrane with normal cell protrusions, as well as an intact nuclear membrane. Organelles such as mitochondria are identifiable (scale bar: 10 μm). **(aii)** Representative image of the cell membrane showing normal cellular protrusions (scale bar: 2 μm). **(bi)** HeLa cell after exposure to tamoxifen (positive autophagy control). Cells displayed vacuolar structures, although the cell membrane, nucleolus and nuclear membrane remained intact. **(bii)** Representative image of a vacuolar structure (scale bars represent 5 μm in **(bi)** and 0.5 μm in **(bii)**). Figure **(ci)** and **(cii)** are representative images of actinomycin D-exposed cells showing apoptotic bodies, pyknosis and an increase in the number of cellular membrane protrusions. The nuclear membrane is absent (scale bars: 5 μm in **(ci)** and 2 μm in **(cii)**). Images **(di)** and **(dii)** of ESE-16-treated cells showed the presence of vacuolar structures (similar to the tamoxifen control), apoptotic bodies, nuclear condensation, increased membrane protrusions and absence of the nuclear membrane demonstrating a cell in distress (scale bars: 5 μm **(di)** and 2 μm **(dii)** respectively). (Arrow colour key: orange = nucleoli; red = nuclear membrane; blue = vacuolar structures; yellow = pyknosis; white = apoptotic bodies).

TEM was used to analyze detailed intracellular and membrane properties of HeLa cells exposed to ESE-16 in order to demonstrate morphological evidence of autophagic and apoptotic cell death, both of which were hypothesized to be caused by ESE-16. Figure 10ai demonstrates the smooth cell membrane with normal cell protrusions (Figure 10aii) of cells exposed to the DMSO vehicle control. Comparative cellular morphology to cells propagated in growth medium was observed (not shown). Intracellular organelles including mitochondria, nucleoli and nuclear membranes are clearly identifiable, with no vacuolar formation. In contrast, cells exposed to the positive control agents, tamoxifen and actinomycin D, displayed characteristics of cellular distress and processes of autophagy and apoptosis respectively. Figure 10bi shows the marked increase of vacuolar structures in response to tamoxifen exposure. These vacuolar structures were most likely autophagosomes in the light of increased LC3 B expression observed as determined by flow cytometry. The latter observation was further supported by an increase in aggresomes demonstrated via a significant AAF value. Cells are smaller in size, and the nuclear membrane, nucleolus and cell membrane are still intact. Figure 10bii shows one of the vacuolar structures (most likely an autophagosome), demonstrating the engulfed cellular contents and organelles within a membrane. Actinomycin D treatment had a pronounced effect on cell membrane structure, with an increase of membrane protrusions (Figure 10ci and cii). Additionally, signs of apoptosis are evident and include pyknosis, karyorhexis and the presence of apoptotic bodies. Figure 10d demonstrates the effect that ESE-16 exposure has on HeLa cells. A significantly distressed cell (representative image of cells treated with ESE-16) in the process of undergoing both apoptosis and autophagy is depicted (Figure 10di). Combination of cell death types I and II are represented by the characteristic apoptotic body formation, chromatin hypercondensation and an increased number of cell membrane protrusions of the former and the presence of numerous vacuolar structures (most likely autophagosomes) as part of the latter. The nuclear membrane is absent. Vacuolar structures and organelles such as Golgi bodies were identified (Figure 10dii).

### Confocal microscopy

**Figure 11:**
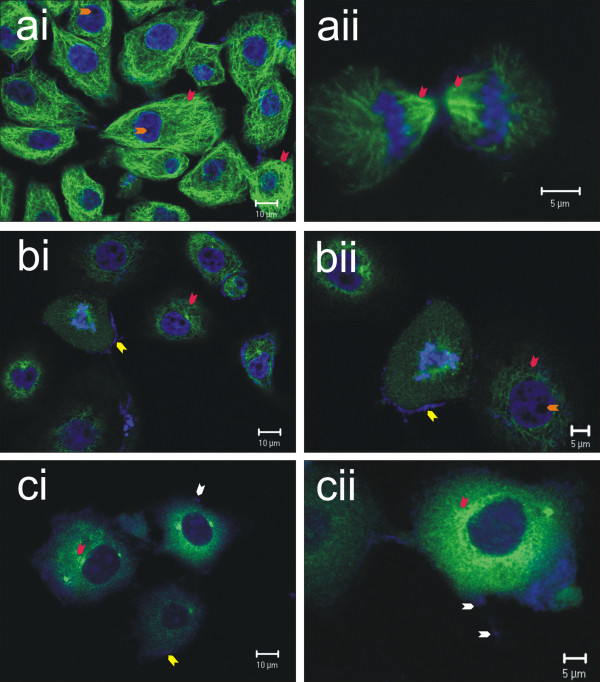
**Confocal microscopic immunofluorescent images of the microtubule structure within ESE-16-exposed HeLa cells.** Alexa-488 fluorescent labeled tubulin-α antibodies fluoresce green allowing visualization of the tubulin network, while DAPI stained nuclear material blue. **(ai)** DMSO exposure (vehicle control) demonstrated cell confluency, various stages of cell division and intact organised tubulin networks as seen in medium only exposed cells (not shown). **(aii)** A cell in telophase, demonstrating intact microskeletal structure. **(bi)** and **(bii)** HeLa cells exposed to 2-ME as a positive control for a microtubule depolymerizing agent, demonstrated compromised cell density and cells in metaphase block with disorganised tubulin structures. **(ci)** and **(cii)** 24 Hour ESE-16 exposure showed complete tubulin disintegration together with apoptotic body formation, DNA margination and a decrease in cell density. (Arrow colour key: orange = nucleoli; yellow = DNA margination; white = apoptotic bodies; red = microtubules, organized and intact in the DMSO vehicle control, and disintegration in the 2-ME control and ESE-16-treated cells).

In order to observe the effects of ESE-16 on HeLa cell cytoskeletal microtubule architecture, confocal microscopy was used to visualize α-tubulin. Figure 11a is a representative image of HeLa cells exposed to the DMSO vehicle control. Confluency of cell growth in various stages of mitosis (mostly interphase) was demonstrated with organized spindle formation visible within these cells. A cell undergoing telophase is shown in Figure 11aii with the chromatids being separated by an organised and functional spindle formation. Cells were exposed to 2-ME as a positive control for spindle depolymerization. Figure 11bi and Figure 5bii demonstrate cells in metaphase block and disorganized tubulin structure after 2-ME exposure with a decrease in cell density. Figure 11c depicts the effect that ESE-16 exposure has on the HeLa cell microskeleton. Total disintegration of the tubulin network is visible in Figure 11ci and cii. Additional evidence of apoptosis was identified in these cells and included margination of DNA, a decrease in cell density and apoptotic body formation.

## Discussion

In order to combine and enhance the anti-cancer characteristics of 2-ME with the advantages gained by strategic modification previously mentioned, ESE-16 was *in silico*-designed. Dose-dependent studies over a 24-hour period were conducted in our laboratory and an IC_50_ of 0.5 μM on HeLa cervical adenocarcinoma cells using the crystal violet staining method was established (data not shown). This showed an increased potency when compared to 2-ME, which decreased HeLa cell numbers to 60% after exposure to 1 μM 2-ME for 72 hours [37,38]. The mechanisms of cell death induced by ESE-16 were further investigated in this study.

Confocal images obtained where HeLa cells were exposed to 0.5 μM ESE-16 for 24 hours and stained with anti-tubulin α antibodies revealed disintegration of tubulin structure, supporting the previous report of microtubule abrogation in MDA-MB-231 cells [33]. These results confirmed ESE-16 as a spindle poison, resulting in the G_2_/M block. Light microscopic morphological changes also revealed a marked decrease in cell density, with the remaining cells being mostly shrunken and rounded. Cellular debris was evident, as well as the presence of apoptotic bodies. Fluorescent- and transmission electron microscopy corroborated characteristics of both apoptosis and autophagy.

Cell cycle progression analysis by flow cytometry of ESE-16-exposed HeLa cells demonstrated a metaphase block. Loss of cell membrane asymmetry occurring with the translocation of phosphatidylserine to the outside of the cell during apoptosis induction (PS-flip) was also quantified with flow cytometry. ESE-16-exposed HeLa cells demonstrated a decrease in cell viability to 64.22%, with a concomitant increase in the number of cells in apoptosis (27.32%). Results confirmed prior data from our laboratory that (200 nM) ESE-16-exposed MDA-MB-231 cells showed a G_2_/M block after 24 hours, as well as an increase in the sub-G_1_ fraction, whereas after 48 hours nearly all the cells were present be in the sub-G_1_ fraction [33].

The induction of mitotic block followed by apoptosis (and autophagy) in MCF-7 cells exposed to 1 μM 2-ME for 24 hours has previously been published by our laboratory [6]. Gene expression microarrays showed differential expression of genes mapped to a variety of processes involved in managing the cell in metaphase block and subsequent programmed death induction (including genes involved in microtubule dynamics, cell cycle checkpoints, cyclin B1 degradation, autophagy and apoptotic regulation) [6].

In the light of the mitotic block demonstrated in HeLa cells exposed to 0.5 μM ESE-16 for 24 hours by morphological studies and flow cytometric analysis, levels of cyclin B1 were determined using flow cytometry. An overall 2.47-fold increase of cyclin B1 was determined when compared to the vehicle control. A higher fluorescent signal was emitted from the smaller more complex cells (representing cells in metaphase block) when fluorescence against side- and forward scatter was plotted. Up-regulation of cyclin B1 expression corroborated the finding of the cell cycle analysis, in which there was a significant increase in cells in metaphase after ESE-16 exposure. Formation of tetrapoidic cells after mitotic spindle inhibition may have caused forced overexpression of cyclin B1, as well as showing a role of this molecule in cell size determination [34,39].

The fate of a cell in unmitigated mitotic arrest seems to depend on the interplay between cyclin B and MCL1 levels [40]. Both MCL1 and cyclin B are at peak concentrations just after spindle poisons have induced the mitotic block: the former to apprehend the onset of apoptosis while cellular restoration is being attempted, and the latter due to the SAC-induced arrest of cell cycle progression due to unattached kinetochores [41]. A rapid up-regulation of cyclin B1 was demonstrated by Newman *et al.* in MCF-7 cells on exposure to 500 nM taxol, 2-ME and 2-MEbisMATE after 24 hours [42]. The increase in cyclin B blocks the progression of the cell cycle and apoptosis until it is degraded, at which point the cell is able to resume its cycle or undergo apoptosis. After 48 hours, the levels of cyclin B deteriorated in the exposed MCF-7 cells, allowing the cells to undergo apoptosis via p53 induction (2-ME did not induce p53 at that concentration) [42]. Additionally, the anti-apoptotic BCL2 protein had been deactivated by phosphorylation in the 2-MEbisMATE and taxol-treated MCF-7 cells after 24 hours of exposure [42].

ESE-16 causes a disrupted spindle assembly and may activate the spindle assembly checkpoint (SAC) resulting in mitotic block and inducing apoptosis [43]. Increased cyclin B1 levels may also be due to ESE-16 blocking the mitotic escape routes downstream of the checkpoint which prevents the premature exit of cells from the induced apoptosis pathways, thereby preventing resistance to the compound’s effects and increasing its anti-tumorigenic properties. The latter serves to slow down proteolytic breakdown of cyclin B1, allowing an increased opportunity for death initiation [43].

2-ME has been implicated in induction of the extrinsic apoptotic pathway in several cell lines [44]. Both caspase 8 and 3 were up-regulated after a 24 hour exposure of HeLa cells to 0.5 μM ESE-16 in this study. Since caspase 3 is an executioner caspase common to both intrinsic and extrinsic pathways, the deduction that ESE-16 induces a caspase-dependent mode of cell death can be made.

Induction of the intrinsic apoptotic pathway with the release of cytochrome *c* causes the formation of the active apoptosome, resulting in the activation of caspase 9, which in turn cleaves the downstream executioner caspases 3, 6 and /or 7 [45]. Mitochondrial membrane potential is affected in ESE-16-exposed HeLa cells indicates involvement of the intrinsic apoptotic pathway. The latter was substantiated by the demonstration of caspase 6 activity [18]. The increase in caspase 8 activity in this study indicates the possibility of an extrinsic pathway concomitantly with the intrinsic pathway.

Evidence of autophagy occurring simultaneously to apoptosis in HeLa cells exposed to 0.5 μM ESE-16 was indicated via MDC fluorescent microscopy and TEM analysis. In order to support these findings, the AAF was calculated in a flow cytometric assay based on the principle that misfolded proteins are relegated to aggresomes which are cleared by autophagy. Additionally, the quantification of autophagy-related protein LC3 B was done in ESE-16 exposed HeLa cells. LC3 B is required for the formation of autophagosomes [46]. Results demonstrated an increase in the AAF, as well as LC3 B expression in ESE-16-treated cells, thus indicating that autophagy is induced along with apoptosis.

Caspases and Beclin-1 may mediate cross talk between apoptosis and autophagy [47]. When Beclin-1, a B-cell lymphoma 2 (BCL2) homology domain 3 (BH3) family member is bound to BCL2 or BCL-extra long (BCL-XL), its interaction with phosphatidylinositol 3-kinase Catalytic Subunit Type 3 (PI3KC3) along with other proteins which are core to the autophagy-inducing complex, is inhibited, thereby preventing autophagy [48,49]. However, Beclin-1 and PI3KC3 are direct substrates of caspases (3, 7 and 8), a process which may be observed during induction of both the intrinsic and extrinsic apoptotic pathway [50]. BCL-2–associated X protein (BAX) over-expression, which induces the intrinsic apoptotic pathway has been shown to cause caspase cleavage of Beclin-1, as does activation of tumor necrosis factor (TNF)-related apoptosis-inducing ligand (TRAIL) [51,52]. Once cleaved, the Beclin-1 C-terminal acquires a new apoptotic-promoting function [50]. Autophagy-related protein 4D (Atg4D) cleavage by caspase 3 induces autophagy activity, but has a cytotoxic effect which amplifies apoptosis via the intrinsic mitochondrial pathway [53]. Thus it can be proposed that caspase cleavage of Beclin-1 and Atg4D can prevent protective autophagic induction and the C-fragment of Beclin-1 can sensitize cells to pro-apoptotic signals [47]. Crosstalk between autophagy and apoptosis in HeLa cells induced by ESE-16 destruction of microtubule integrity and resultant metaphase block, involves the extrinsic pathway with increased caspase 8 and 3 activity.

## Conclusion

Data from this *in vitro* study supports the concept that the novel *in silico*-designed estradiol analogue, ESE-16, may act as a potential anti-cancer drug. ESE-16 induced both autophagy and apoptosis in cervical adenocarcinoma (HeLa) cells in response to a persistent mitotic block caused by microtubule abrogation. An increase in caspases 8 and 3 implicated the extrinsic pathway of induction. The involvement of Beclin-1 as a mediator between autophagy and apoptotis in ESE-16 exposed cells must be established. Future *in vivo* studies will determine whether this novel anti-neoplastic drug exerts any significant side-effects and whether the *in silico*-design of our laboratory to increase the compounds’ bioavailability was successful.

## Materials and methods

### Reagents

Dulbecco’s Modified Eagle Medium (DMEM) was purchased from Separations (Johannesburg, South Africa). European-grade heat-inactivated fetal calf serum (FCS) and RNase A were obtained from BIOCOM biotech (Pty) Ltd. (Clubview, South Africa). Syringe filters (0.22 μm), sterile cell culture flasks and plates were obtained through Sterilab Services (Kempton Park, Johannesburg, South Africa). Penicillin, streptomycin and fungizone were purchased from Highveld Biological (Pty) Ltd. (Sandringham, South Africa), as was the trypsin/versene. Triton X-100, 100% ethanol, and propidium iodide were purchased from Sigma-Aldrich (St. Louis, Missouri, United States of America (USA). All additional chemicals were of analytical grade and were purchased from Sigma-Aldrich (St Louis, Missouri, USA).

### Chemical compound and appropriate controls

The non-commercially available 2-ethyl-3-*O*-sulphamoyl-estra-1,3,5(10)16-tetraene (ESE-16) was synthesized by iThemba (PTY) Ltd. Pharmaceuticals (Modderfontein, Gauteng, South Africa). The IC_50_ of ESE-16 in HeLa cells was established to be 0.5 μM via a crystal violet staining technique in a dose-dependent study (data shown in Additional file 1). As a negative vehicle control, medium of the cell samples were supplemented with an equal concentration of dimethyl sulphoxide (DMSO). The DMSO content of the final dilutions never exceeded 0.1% (v/v). Positive controls constituted actinomycin D at a final concentration of 0.1 μg/ml for apoptosis, 20 μM tamoxifen for autophagy and 1 μM 2-ME for microtubule depolymerization (Sigma-Aldrich, St Louis, Missouri, USA).

### Cell line and cell culture

The commercially available tumorigenic human epithelial cervical cell line, namely the HeLa cell line, was acquired from Highveld Biological (Pty) Ltd (Sandringham, Johannesburg, South Africa). HeLa cells were propagated and maintained in 25 cm^2^ or 75 cm^2^ tissue culture flasks in a humidified atmosphere at 37°C, 5% CO_2_ in a Forma Scientific water-jacketed incubator (Ohio, USA). HeLa cells were cultured in DMEM supplemented with 10% heat-inactivated FCS (56°C, 30 minutes) for optimal cell growth. 100 U/ml penicillin G, 100 μg/ml streptomycin and fungizone (250 μg/L) were added to the medium for infection control. Experiments were performed in either six-well plates or 25 cm^2^ cell culture flasks. For six-well plates, exponentially growing cells were seeded at 350 000 cells per well in 3 ml fresh maintenance medium. For 25 cm^2^ cell culture flasks, exponentially growing HeLa cells were seeded at 1 X 10^6^ cells per flask to a final volume of 5 ml of maintenance medium. After a 24-hour incubation period at 37°C to allow for cell adherence, cells were exposed to 0.5 μM ESE-16 and the relevant controls were included. Cells were incubated for a further 24 hours at 37°C as the exposure time-frame.

### Cell cycle analysis

Exposed cells were trypsinized and collected for analysis. Cells were centrifuged at 150 *g* and subsequently resuspended in 200 μl ice-cold PBS containing 0.1% FCS. Ice-cold 70% ethanol was added in a drop-wise manner while vortexing gently. Samples were incubated at 4°C for a minimum of 12 hours, after which the cells were pelleted by centrifugation. Cells were resuspended in PBS containing propidium iodide (40 μg/ml), RNase A (100 μg/ml) and Triton X-100 (0.1%) and incubated while protected from light at 37°C for 40 minutes. Analysis entailed measurement of propidium iodide fluorescence (FL3) on a FC500 system flow cytometer (Beckman Coulter, South Africa (PTY) Ltd.) equipped with an air-cooled argon laser excited at 488 nm. Aneuploid and aggregated cells, as well as cell debris were gated out by visual inspection. Cell cycle distributions from generated histograms were expressed as a percentage of cells in each phase.

### Apoptosis studies

Analysis was performed using the BioVision Annexin V-FITC reagent kit acquired from BioVision Research Products (Mountain view, California, USA. The 1X binding buffer (supplied in the kit) was prepared as directed. According to manufacturer’s protocol, 500 000 exposed cells from each flask were washed in PBS, centrifuged and resuspended in 1X binding buffer. Double staining was done by adding 1 μl Annexin V-FITC and 1.2 μl 40 μg/ml propidium iodide to the cells, which were incubated for five minutes at room temperature in the dark. Annexin V (FL1) and propidium iodide (FL3) fluorescence were measured in 10 000 cells per sample with a FACS FC500 System flow cytometer (Beckman Coulter South Africa (Pty) Ltd.) equipped with an air-cooled argon laser excited at 488 nm. Results were expressed as a percentage of cells in three categories namely; viable cells, apoptotic cells and necrotic cells.

### Cyclin B1 detection: metaphase block

Exposed cells were trypsinized, collected, centrifuged and washed with 1 ml ice-cold PBS. Cells were resuspended in 200 μl ice-cold PBS containing 0.1% FCS. Cells were fixed with 10 ml ice-cold 70% ethanol and stored at 4°C for 24 hours. Cells were centrifuged and washed twice with PBS to remove the ethanol. A working antibody solution (Milli-Mark™ Anti-Cyclin B1-PE, clone GNS3 (8A5D12), Millipore Corporation, Temecula, California, USA) was prepared by diluting the primary antibody in PBS in a 1:5 ratio. A 1:10 antibody solution to 0.1% Triton X-100 PBS was added per 1 X 10^6^ cell sample. Cells were incubated in the conjugated cyclin B1 antibody solution for 40 minutes at 37°C. Cells were washed with PBS. All procedures after the incubation with the antibody were conducted in a light protected environment. PE (FL3) fluorescence was measured with a FC500 System flow cytometer (Beckman Coulter South Africa (Pty) Ltd.) equipped with an air-cooled argon laser excited at 488 nm. Aggregated and aneuploid cells were excluded from the analysis. 10 000 cells were analyzed per sample. Measurement of PE-conjugated cyclin B1 fluorescence of control and exposed HeLa cells was done using the normalized area of the dot-plot.

### Autophagy: aggresome detection

The Enzo Life Sciences’ ProteoStat® Aggresome Detection Kit was purchased from Enzo Life Sciences Inc. (New York, USA). The kit provided the ProteoStat® Aggresome Detection Reagent and the 10X assay buffer. Subsequent to the 24 hour exposure, cells were harvested via trypsinization and resuspended in 200 μl PBS prior to adding 4% formaldehyde in a drop-wise manner while vortexing the tube. Cells were left at room temperature for 30 minutes. Samples were centrifuged and a PBS washing step followed. While vortexing, permeabilizing solution was added in a drop-wise fashion, after which the sample was incubated for 30 minutes on ice. Cells were collected by centrifugation, after which they were washed in PBS. Cells were resuspended diluted ProteoStat® Aggresome detection reagent. Cells were incubated at room temperature for 30 minutes in the dark. Fluorescence within 10 000 cells of each sample was analyzed in the FL3 channel of the FACS FC500 System flow cytometer (Beckman Coulter, South Africa (Pty) Ltd.) equipped with an air-cooled argon laser excited at 488 nm. After obtaining the mean fluorescence intensity (MFI) for the samples (cells propagated in medium only as the negative control, ESE-16-treated cells and a positive autophagy control), the aggresome activity factor (AAF), as determined according to the supplier’s manual, was calculated as follows:

$$\text{AAF}=100^{*}\left({\text{MFI}}_{\text{Rx}}-{\text{MFI}}_{\text{CONT}}\right)/{\text{MFI}}_{\text{Rx}}$$

where AAF is the aggresome activity factor; MFI_Rx_ is the mean fluorescence intensity of the treated sample; and MFI_CONT_ is the mean fluorescence intensity of the control sample. An AAF of more than 25 was indicative of a positive result and a significant increase in aggresome formation.

### Autophagy-related protein LC3 B determination

After a 24 hour exposure to ESE-16 and the relevant controls, cells were trypsinized and washed with ice-cold PBS. Cells were fixed with 0.01% paraformaldehyde/PBS for 10 min, then pelleted and resuspended in ice-cold PBS. Cells were permeablized with ice-cold methanol (−20°C), added in a drop-wise manner. Cells were washed twice with cold PBS. Pelleted cells were resuspended in the primary antibody solution (0.5:1000 MAP1LC3 B rabbit antibody (Biosensis, Thebarton, Australia) to PBS, 1% BSA) and incubated for 2 hours at 4°C protected from light. Cells were washed thrice with washing buffer (PBS, 0.05% Triton, 1% BSA). Blue carrier protein (FL1) fluorescence was measured with a FC500 System flow cytometer (Beckman Coulter SA (Pty) Ltd.) equipped with an air-cooled argon laser excited at 488 nm. Data from at least 10 000 cells were analyzed. Measurement of Blue Carrier Protein-conjugated MAP1LC3 B antibody fluorescence of control and exposed HeLa cells was done, using the normalized area of the dot-plot. An overlay histogram was constructed from the dot plots using Cyflogic version 1.2.1 software (Pertu Therho, Turko, Finland).

### Quantification of caspases 8 and 3

The FLICE/Caspase 8 colorimetric kit was used to determine caspase 8 activation and the BioVision Caspase-3/CPP32 Colorimetric Assay Kit was used to quantify caspase 3 activity. Both kits were purchased from BioVision Research Products (Mountain View, California, USA). Included were the cell lysis buffer, 2X reaction buffer, 4 mM IETD-*p*NA (in the caspase 8 kit), 4 mM DEVD-*p*NA (in the caspase 3 kit), 1 M dithiothreitol (DTT) and the dilution buffer. 1–5 × 10^6^ treated cells were pelleted by centrifugation after trypsinization, and resuspended in ice-cold cell lysis buffer and incubated on ice for 10 minutes. Following centrifugation, the supernatant was transferred to eppendorf tubes and placed on ice. The protein concentration was determined using the Pierce® BCA protein assay kit (Thermo Fisher Scientific Inc. Rockford, Illinois, USA) as per manufacturers protocol. Protein (50 μg) of each assay was diluted in 50 μl cell lysis buffer and placed in a 96-well plate, to which an equal volume of 2× reaction buffer (containing 10 mM DTT) was added. The reaction was initiated by adding 5 μl DEVD-*p*NA for caspase 3 or IETA-*p*NA for caspase 8. Plates were incubated for 120 minutes at 37°C, protected from light and the absorbance was read at 405 mn on the BioTek Epoch multi-volume spectrophotometer system (BioTek Instruments Inc., Analytical Diagnostic Products, Weltevreden Park, South Africa). Background readings from the buffers were first subtracted from the total readings, after which the fold increase of caspase activity was determined by using the DMSO vehicle control as the baseline reading.

### Polarization-optical transmitted light differential interference contrast microscopy

Viable cells (3.5 × 10^5^) determined by trypan blue exclusion, were seeded in six-well plates and allowed to attach for 24 hours. PlasDIC images were obtained using the Zeiss Axiovert-40 microscope (Göttingen, Germany) and Zeiss Axiovert MRm monochrome camera (Carl Zeiss MicroImaging GmbH, Göttingen, Germany) before and after a 24 hour exposure to vehicle controls, positive controls or ESE-16 respectively.

### Fluorescent microscopy

Fluorescent microscopy using monodansylcadaverine (MDC) was employed to determine the effect that ESE-16 has on acidic vesicular organelle formation [36]. After discarding the growth medium, ESE-16 exposed cells (and relevant controls) were incubated in 0.05 mM MDC for 10 minutes at 37°C. Cells were washed four times with PBS. Cells were examined with a Zeiss inverted Axiovert CFL40 microscope and Zeiss Axiovert MR monochrome camera (Carl Zeiss (Pty) Ltd., Johannesburg, South Africa). A UV filter with excitation 380 nm and emission 420 nm was used for detection of MDC staining. In order to curtail fluorescent dye quenching, all procedures were performed in a dark room.

### Transmission electron microscopy

After a 24 hour exposure to ESE-16 and the relative controls, cells were collected via trypsinization and fixed in diluted Karnovsky’s fixative (2.5% glutaraldehyde and 2.5% formaldehyde in 0.075 M phosphate buffer at pH 7.4–7.6) for 45 minutes at room temperature. Cells were washed with 0.075 M phosphate buffer. Cells were fixed in 0.5% aqueous osmium for two hours, followed by a rinsing step with distilled water. Dehydration steps followed and entailed increasing concentrations of ethanol (30%, 50%, 70%, 90%, 100%, 100%, 100%). Cells were infiltrated with a series of increasing percentage of quetol epoxy resin (30% for 30 minutes, 60% for 30 minutes and 100% quetol for four hours). Embedding of samples followed and polymerization of the specimens was allowed for 36 hours at 60°C. Ultra-thin sections were then prepared with a microtome and mounted on a copper grid. Samples were contrasted with 4% aqueous uranyl acetate and Reynolds’ lead citrate. All chemical were obtained from Merck (Darmstadt, Germany). Samples were viewed with a JEM-210 °F field emission transmission electron microscope (JEOL, Tokyo, Japan) at the Laboratory for Microscopy and Microanalysis, University of Pretoria (Pretoria, South Africa).

### Confocal microscopy

Exponentially growing HeLa cells were seeded at 350 000 cells per well in 6-well plates on flame-sterilized cover slips. After exposure, cells on cover slips were washed with pre-warmed cytoskeletal buffer (CB) (60 mM PIPES, 27 mM, 10 mM EGTA and 4 mM magnesium sulphate with the pH adjusted to 7.0 with NaOH). Fixation of cells was done with in pre-warmed 0.3% gluteraldehyde for 10 minutes at 37°C. This was followed by a wash step with warmed cytoskeletal buffer while agitating. Cell membranes were permeablized using the permeabilization buffer (1% Triton X-100 in CB) and agitated for 15 minutes. Cells were washed once with CB buffer and then washed twice with phosphate-buffered saline (PBS). Non-reacting aldehydes were removed by treating samples three times with the reducing agent (5 mg sodium borohydrite in 5 ml PBS). Cover slips were incubated in a 10% normal host serum-blocking buffer (10% FCS in PBS containing 0.05% Triton X-100) for 60 minutes. Cover slips were incubated in a primary mouse anti-tubulin alpha antibody (monoclonal Antibody to Tubulin-alpha (clone DM1A), Imgenex, San Diego, California, USA) cocktail (1:100 ratio antibody to PBS-Triton wash buffer diluted with 50% blocking buffer) for 90 minutes at 37°C in a humidity chamber. Cover slips were washed at room temperature with PBS-Triton wash buffer (PBS with 0.05% Triton X-100) with 1% FCS. Samples were incubated while protected from light for 90 minutes at 37°C after adding secondary fluorescent labelled (alexa Fluor® 488 dye) anti-mouse antibodies (raised in donkey) (Invitrogen, Paisley, United Kingdom) cocktail (1:125 as above) with a final concentration 2 mg/ml. Samples were washed with PBS-Triton wash buffer with FCS. Nuclear staining was achieved by incubating the cover slips in 50% DAPI (Thermo Fisher Scientific Inc. Rockford, Illinois, USA) to equal volumes of PBS for five to ten minutes while protected from light. Counterstained cover slips were then washed with distilled water, mounted with a glycerol-based mounting fluid and cells were examined with a Zeiss LSM 510 Meta Confocal Microscope (Zeiss, Jena, Germany) at the Laboratory for Microscopy and Microanalysis, University of Pretoria, Pretoria, South Africa. Cells were visualized using immunofluorescent techniques in which green emissions from the Alexa-488 probe were collected at 519 nm (excited at 495 nm) and the blue DAPI at 461 nm (excited at 358 nm).

### Statistical analysis

Quantitative data were obtained from three independent experiments. In addition, each of the independent experiments was conducted in three replicates and is shown as the mean ± standard deviation (SD). Quantitative data were statistically analyzed for significance using the analysis of variance (ANOVA)-single factor model followed by a two-tailed Student’s *t*-test. Means are presented in bar charts, with T-bars referring to standard deviations. *P*-values of less than 0.05 were regarded as statistically significant and are indicated by an asterisk (*). For flow cytometry, three independent experiments were done in triplicate and data from at least 10 000 cells were analyzed using Cyflogic version 1.2.1 software (Pertu Therho, Turko, Finland). Flow cytometric measurement of cell cycle progression and protocols involving FITC-labels and the aggresome kit are expressed as relative fluorescence (a percentage of the value measured for vehicle-treated negative control cells). Morphological microscopy studies (qualitative data) were repeated three times.

## Abbreviations

2-ME: 2-methoxyestradiol; ESE-16: 2-ethyl-3-*O*-sulphamoyl-estra-1,3,5(10)16-tetraene; CA: carbonic anhydrase; DMSO: Dimethyl sulfoxide; Act D: Actinomycin D; PI3KC3: phosphatidylinositol 3-kinase Catalytic Subunit Type 3; BCL2: B-cell lymphoma 2; TRAIL: tumor necrosis factor (TNF)-related apoptosis-inducing ligand; MDC: monodansylcadaverine.

## Competing interests

The authors declare no competing interests.

## Authors’ contributions

AT, EN, LL and AJ were involved in the concept and design, analysis and interpretation of the data and drafting of the manuscript. AT and EN performed all the experiments and acquired the data. All authors read and approved the final manuscript.

## Supplementary Material

Additional file 1 Dose response curve: Cytotoxicity determination. Click here for file
