# Supplementary material for: Molecular crosstalk between apoptosis and autophagy induced by a novel 2-methoxyestradiol analogue in cervical adenocarcinoma cells
Source: Cancer Cell Int. 2013 Aug 27;13:87. doi: 10.1186/1475-2867-13-87 (PMC3766685; doi:10.1186/1475-2867-13-87)
Supplement: Additional file 1 — Dose response curve: Cytotoxicity determination. [file 1475-2867-13-87-S1.docx]

**Dose response curve: Cytotoxicity determination**

Method

The number of viable cells after 24 hour exposure to ESE-16 was determined using the crystal violet method, which stains DNA [[1](#_ENREF_1)]. A dose-dependent study was conducted using a concentration range of 0.5 - 25μM. HeLa cells (5 000 cells per well) were seeded in 96 well tissue culture plates and incubated at 37°C in a humidified atmosphere (5% CO_2_) for 24 h to allow for attachment. To establish the starting number of cells, a baseline determination was conducted before exposure. Cells were exposed ESE-16 at a concentration series of 0.5 - 25μM for the 24 h at 37^o^C. DMSO vehicle-treated controls were also included. Cells were fixed with 100µl of 1% gluteraldehyde and incubated for 15 minutes). Cells were incubated in 100µl 0.1% crystal violet for 30 minutes. The 96 well plate was rinsed under running water. Cells were detached using 200µl 0.2% Triton X-100 at room temperature for 30 minutes and transferred (100µl) to a new microtitre plate. Absorbance was determined at 570nm using an EL_x_800 Universal Microplate Reader (Bio-Tek Instruments Inc. (Vermont, United States of America)).

Results

Dose-dependent studies were conducted with the purpose of evaluating the antiproliferative effects of ESE-16 in HeLa cells after 24 h of exposure. The concentration at which 50% ± 4.6 of the cells survived (IC_50_) after ESE-16-exposure over for 24 hours was calculated to be 0.5 µM (Figure 1).


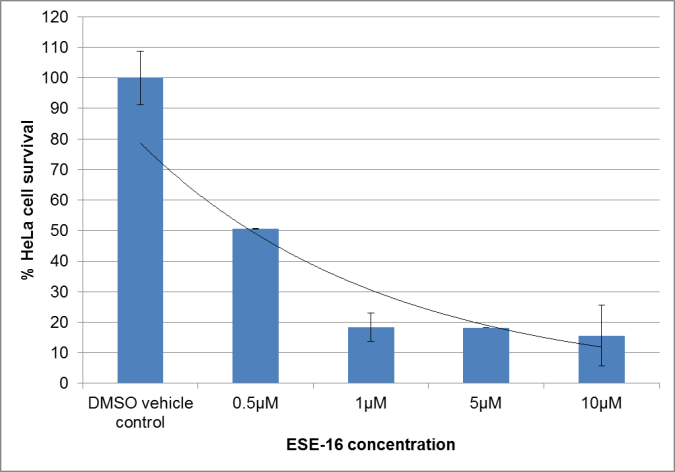

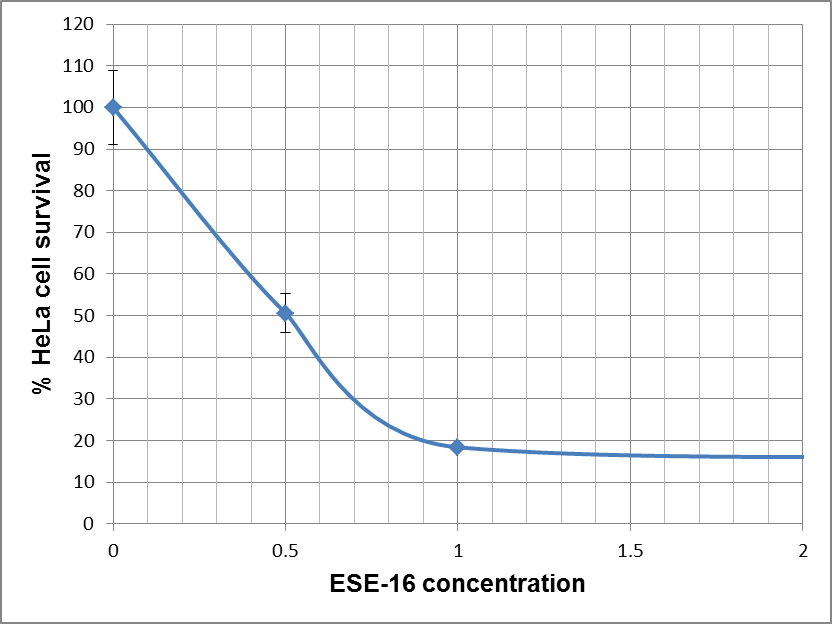


Figure 1: Dose response curve of HeLa cells to ESE-16. Viable HeLa cell numbers were expressed as a % of cells relative to the DMSO vehicle control exposure to ESE-16 for 24 hours. Standard deviation represented by T-bars.

References

1. Gillies RJ, Didier N, Denton M: **Determination of cell number in monolayer cultures**. *Analytical biochemistry* 1986, **159**(1):109-113.
